# Probing Transcriptional Crosstalk between *Cryptochromes* and *Iron-sulfur Cluster Assembly 1* (*MagR*) in the Magnetoresponse of a Migratory Insect

**DOI:** 10.3390/ijms241311101

**Published:** 2023-07-05

**Authors:** Yuning Zhang, Ying Zhang, Jingyu Zhao, Jinglan He, Zongjin Xuanyuan, Weidong Pan, Gregory A. Sword, Fajun Chen, Guijun Wan

**Affiliations:** 1 Department of Entomology, College of Plant Protection, Nanjing Agricultural University, Nanjing 210095, China; 2022102093@stu.njau.edu.cn (Y.Z.); 2021102064@stu.njau.edu.cn (Y.Z.); 2016102054@njau.edu.cn (J.H.); fajunchen@njau.edu.cn (F.C.); 2 Key Laboratory of Integrated Pest Management on Crops in East China, College of Plant Protection, Nanjing Agricultural University, Nanjing 210095, China; 3 Beijing Key Laboratory of Bioelectromagnetics, Institute of Electrical Engineering, Chinese Academy of Sciences, Beijing 100190, China; panwd@mail.iee.ac.cn; 4 Department of Entomology, Texas A&M University, College Station, TX 77843, USA; gasword@tamu.edu

**Keywords:** migratory insect, cryptochrome (Cry), *Iron-sulfur Cluster Assembly 1 (MagR)*, magnetoresponse, magnetoreception, geomagnetic field, near-zero magnetic field, brown planthopper

## Abstract

Many organisms can sense and respond to magnetic fields (MFs), with migratory species in particular utilizing geomagnetic field information for long-distance migration. Cryptochrome proteins (Crys) along with a highly conserved Iron-sulfur cluster assembly protein (i.e., MagR) have garnered significant attention for their involvement in magnetoresponse (including magnetoreception). However, in vivo investigations of potential transcriptional crosstalk between *Crys* and *MagR* genes have been limited. The brown planthopper, *Nilaparvata lugens*, is a major migratory pest insect and an emerging model for studying MF intensity-related magnetoresponse. Here, we explored in vivo transcriptional crosstalk between *Crys* (*Cry1* and *Cry2*) and *MagR* in *N. lugens*. The expression of *Crys* and *MagR* were found to be sensitive to MF intensity changes as small as several micro-teslas. Knocking down *MagR* expression led to a significant downregulation of *Cry1,* but not *Cry2*. The knockdown of either *Cry1* or *Cry2* individually did not significantly affect *MagR* expression. However, their double knockdown resulted in significant upregulation of *MagR*. Our findings clearly indicate transcriptional crosstalk between *MagR* and *Crys* known to be involved in magnetoresponse. This work advances the understanding of magnetoresponse signaling and represents a key initial step towards elucidating the functional consequences of these novel in vivo interactions.

## 1. Introduction

Many organisms, ranging from microbes to vertebrates, possess the ability to sense and respond to magnetic fields (MFs) [1,2,3], with migratory species in particular utilizing geomagnetic field (GMF) information for navigation or orientation [4,5,6]. The conservation of magnetoresponse across taxa underscores its biological significance, and studies on the topic have experienced substantial growth over the past two decades [6,7,8,9,10,11,12]. With respect to elucidating the mechanisms underlying magnetoresponse, magnetite-based mechanisms [1,13], the radical-pair quantum model [11,14], and, more recently, iron-sulfur cluster assembly (IscA, i.e., MagR)/Cryptochrome (Cry) complex magnetosensing [15,16,17] have been the focus of attention, particularly with regards to the latter two.

In animals, Crys play crucial roles in the circadian function and can be divided into three categories: *Drosophila*-like type 1 Crys, mammalian-like type 2 Crys, and bird-like type 4 Crys (Cry4s) [18]. Type 1 Crys (Cry1s) are UV-A/blue-light photoreceptors responsible for the synchronization of the circadian clock to the daily light-dark cycle [19,20] in most insects, but are absent in vertebrates. Type 4 Crys are light-sensitive, but currently have no known roles in clock function [21]. Type 2 Crys (Cry2s) are light-insensitive and function as circadian transcriptional repressors. They are present not only in mammals but also in most insects, except for flies in the brachyceran lineage [20,22,23]. The canonical radical-pair model proposes that magnetoreception is achieved through quantum effects of magnetically-sensitive flavin-tryptophan radical pairs formed by photosensitive Cry1 and Cry4 [6,8,11,14], although Cry2 has also been found critical in magnetoresponses of several insect species recently [24,25,26]. As technology continues to advance and magnetoresponse research deepens, the canonical biophysical model is evolving. This mainly includes a shift from three to four successive flavin-tryptophan radical pairs [8] and a possible transition from Cry-centric radical-pair mechanisms towards a non-Cry-dependent one [7,27].

Iron-sulfur proteins are widely recognized for their crucial roles in many fundamental physiological processes including cellular respiration, nitrogen fixation, photosynthesis, DNA replication, and repair [28,29,30,31,32]. As a highly conserved A-type iron-sulfur protein, IscA has been proposed as a magnetoreceptor renamed MagR [15,33], which has also been suggested to influence circadian rhythms in *Drosophila* [34]. Research into MagR’s role in magnetoresponse and its potential applications is expanding [16,25,26,35,36,37,38,39,40,41,42,43,44,45,46,47,48,49]. Although no protein interactions between MagR and Cry were observed in the European robin, *Erithacus rubecula* [50], a well-known migrant bird species, such interactions have been identified in other vertebrates and invertebrates. These include pigeon *Columba livia*, human *Homo sapiens*, the brown planthopper, *Nilaparvata lugens* [39], *D. melanogaster* flies, and *Danaus plexippus* butterflies [15], indicating a remarkable degree of conservation. Increasing transcriptional evidence from microorganisms [51], plants [45], and insects [25,36,40,48,49,52] also support MagR’s function in magnetoresponse signaling. Notably, research on migratory insects has provided unique insights, suggesting that MagR may play a role in regulating migration through magnetoreception-related processes [26,40,49]. However, some fundamental questions remain and require further investigation, such as details regarding the origin of MagR’s magnetism [16] and its interaction with Cry.

In recent years, insects have increasingly been utilized in magnetoresponse studies due to their short generation time and the availability of a simple yet powerful genetic toolbox. For example, *D. melanogaster* has been frequently employed to verify the Cry-involved radical-pair quantum mechanism through various assays including food rewarding-based assay [53,54], geotaxis assay [55,56], circadian magnetoresponse assay [7,57,58], and electrophysiology assay [7]. The brown planthopper, *N. lugens*, is a major migratory pest of rice that employs a partial seasonal migration strategy [59]. Its northward migration comprises multiple waves from spring to almost the end of summer, each lasting for one to several days. Upon arrival in new areas, it reproduces, and its migratory offspring continue the next wave of migration. As *N. lugens* is unable to survive winter in temperate East Asia, it undertakes a southward return migration along similar routes during autumn. A series of studies has been conducted to explore the magnetoresponses of *N. lugens* to variations in GMF intensity [38,40,60,61,62,63,64]. In addition, magnetite-based magnetoreception [65] and putative key magnetoresponse genes including *Cry1*, *Cry2*, and *MagR* in *N. lugens* have been investigated [38,40,61,62,66], establishing it as an emerging model for magnetoresponse research.

Recently, there have been increasing reports of in vivo reverse-genetic magnetoresponse studies targeting potential magnetoreceptor genes across taxa [6,7,24,25,26,67]. However, the potential crosstalk between MagR and Cry(s) has not been fully considered, which may introduce bias in functional identification. Here, we investigate the transcriptional interactions between *Crys* (*Cry1* and *Cry2*) and *MagR* in magnetoresponse using an RNAi assay with the migratory *N. lugens*. We show that transcript expression of all three putative magnetoreceptor genes can respond to changes in MF intensity. Notably, there exists a transcriptional interplay between them, indicating that they sense or transduce magnetoresponse in the same signaling pathway.

## 2. Results

### 2.1. Transcriptional Responses of the Putative Magnetosensing Genes to Magnetic Field Intensity Changes

*Nilaparvata lugens* typically undergoes at least one generation of breeding before migrating again to a new site, and unmated 2-day-old adults begin to take off for a nocturnal migration [68,69]. Furthermore, considering the absence of identified magnetosensory organs in this species, this study began by examining the transcriptional responses of potential magnetoreceptor genes (*Cry1*, *Cry2*, and *MagR*) in whole unmated female adults aged 1–3 days old. These individuals were exposed to either a physiological geomagnetic field of 45 μT (GMF_45μT_) or a non-physiological near-zero magnetic field (NZMF) for one generation, compared to the local GMF intensity of 50 μT (GMF_50μT_), before transcript expression analyses.

**Figure 1:**
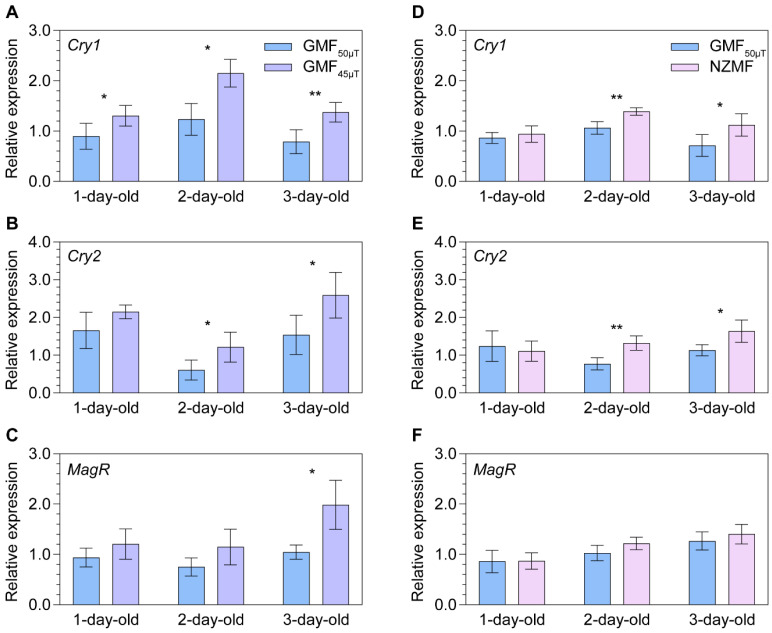
The relative transcript abundance of putative key genes *Cryptochromes* (*Cry1* and *Cry2*) and *Iron-sulfur Cluster Assembly 1* (i.e., *MagR*) in magnetoresponse signaling of one- to three-day-old female adult *Nilaparvata lugens* under the GMF_50μT_ vs. GMF_45μT_ (panels (**A**–**C**)) and the GMF_50μT_ vs. near-zero magnetic field vs. (NZMF) (panels (**D**–**F**)). Statistical significance of expression differences between magnetic fields is tested using one-way ANOVA or two-tailed Mann–Whitney *U* test at *p* < 0.05 (*) and *p* < 0.01 (**).

Transcript expression levels of three putative magnetosensing genes in adult female *N. lugens* were found to be upregulated when the insects were exposed to GMF_45μT_ along their migratory route, as compared to the local GMF_50μT_, for one generation. Significant upregulation of *Cry1* expression was observed in 1-day- (+45.0%, *p* = 0.049), 2-day- (+74.2%, *p* = 0.029), and 3-day-old (+74.1%, *p* = 0.009; Figure 1A) female adults. Transcript expression of *Cry2* was significantly upregulated in 2-day- (+99.5%, *p* = 0.044) and 3-day-old (+68.2%, *p* = 0.039; Figure 1B) female adults, while significantly upregulated *MagR* was only found in 3-day-old (+90.0%, *p* = 0.029; Figure 1C) female adults. Only a marginal upregulation of *MagR* was found in 2-day-old (+52.8%, *p* = 0.092; Figure 1C) female adults.

When *N. lugens* was exposed to NZMF vs. GMF_50μT_, significant upregulation of putative magnetosensing genes in both 2-day- and 3-day-old female adults was found for *Cry1* (+30.5%, *p* = 0.004; +57.3%, *p* = 0.039; Figure 1D) and *Cry2* (+71.0%, *p* = 0.004; +44.6%, *p* = 0.022; Figure 1E), but not for *MagR* (Figure 1F).

### 2.2. Transcript Expression of Cryptochromes after Knocking down the Putative Magnetoreceptor Iron-sulfur Cluster Assembly 1 (MagR)

The RNAi assay was conducted with 2-day- and 3-day-old individuals under a local GMF_50μT_ since the significant differences between magnetic field groups were mainly found in female adults of that age range. The effectiveness of RNA interference targeting individual genes (*Cry1, Cry2*, and *MagR*) and double knockdown of *Cry1* and *Cry2* (*Cry1/2*) was validated before investigating transcriptional interplay, as demonstrated in Figure S1.

**Figure 2:**
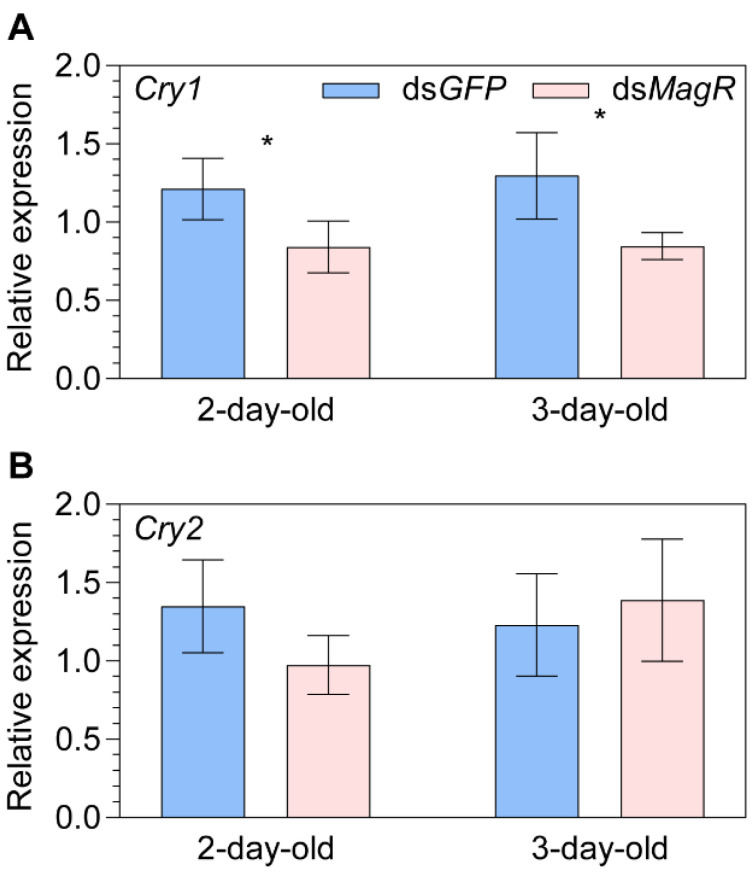
The relative transcript abundance of *Cryptochromes* (**A**) *Cry1* and (**B**) *Cry2* in two- and three-day-old female adult *Nilaparvata lugens* after knocking down of *Iron-sulfur Cluster Assembly 1* (i.e., *MagR*) by ds*MagR* injection. Statistical significance of expression differences between ds*MagR* and ds*GFP* control treatment groups is tested using one-way ANOVA or two-tailed Mann–Whitney *U* test at *p* < 0.05 (*).

There was a significant downregulation of *Cry1* relative to the control ds*GFP* treatment in both 2-day- (−30.6%, *p* = 0.028) and 3-day-old (+34.7%, *p* = 0.029) females after the knockdown of *MagR* (Figure 2A). However, only a marginal downregulation was observed for *Cry2* in 2-day-old females (−27.8%, *p* = 0.077; Figure 2B) and the difference was negligible in 3-day-old females (Figure 2B).

### 2.3. Transcript Expression of the Putative Magnetoreceptor Iron-sulfur Cluster Assembly 1 after Knocking down Cryptochrome 1 or Cryptochrome 2

**Figure 3:**
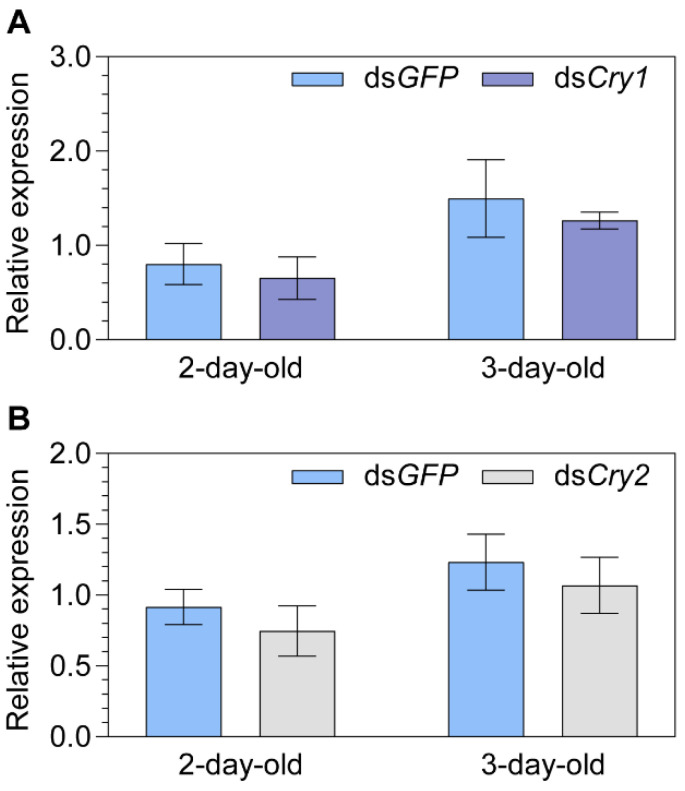
The relative transcript abundance of *Iron-sulfur Cluster Assembly 1* (i.e., *MagR*) in two- and three-day-old female adult *Nilaparvata lugens* after injection of either (**A**) ds*Cry1* or (**B**) ds*Cry2*. Statistical significance between the target gene and ds*GFP* control group expression of *MagR* is tested using one-way ANOVA or two-tailed Mann–Whitney *U* test at *p* < 0.05. No significance was found.

*Cry1* and *Cry2* were individually knocked down to explore the effects on the transcript expression of *MagR*. However, no significant difference in *MagR* expression compared to the ds*GFP* control group was found for either gene (ds*Cry1* vs. ds*GFP*; ds*Cry2* vs. ds*GFP*; *p* ≥ 0.169; Figure 3).

### 2.4. Potential Transcriptional Interactions between Cryptochrome 1 and Cryptochrome 2

To address potential transcriptional interactions between *Cry1* and *Cry2* that could mask the effect of *Crys*’ knockdown on the transcript expression of *MagR*, we evaluated the transcript expressions of both *Crys* individually by knocking down the other *Cry*.

**Figure 4:**
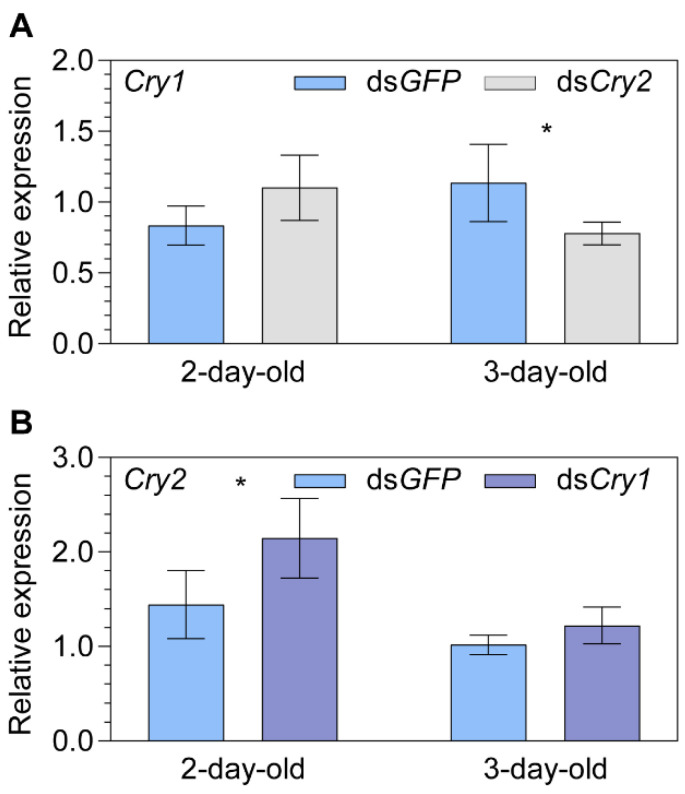
The relationship between *Cryptochrome 1* (*Cry1*) and *2* (*Cry2*) expression levels explored by dsRNA injections. (**A**) The relative transcript expression of *Cry1* in two- and three-day-old female adult *Nilaparvata lugens* after injection of ds*Cry2*. (**B**) The relative transcript expression of *Cry2* in two- and three-day-old female adult *Nilaparvata lugens* after injection of ds*Cry1*. Statistical significance between the target gene and ds*GFP* control group expression levels is tested using one-way ANOVA or two-tailed Mann–Whitney *U* test at *p* < 0.05 (*).

A significant downregulation of *Cry1* was found for 3-day-old females (−31.3%, *p* = 0.029; Figure 4A) in the ds*Cry2* vs. ds*GFP* group, while a significant upregulation of *Cry2* was found for 2-day-old females (+48.6%, *p* = 0.044; Figure 4B) in ds*Cry1* vs. ds*GFP* group. Moreover, a marginal upregulation of *Cry1* was found for 2-day-old females (+32.1%, *p* = 0.093; Figure 4A) in the ds*Cry2* vs. ds*GFP* group. No significance was found between ds*Cry1* vs. ds*GFP* group for the 3-day-old females (*p* = 0.110; Figure 4B).

### 2.5. Transcript Expression of the Putative Magnetoreceptor Iron-sulfur Cluster Assembly 1 after Knocking down Both Cryptochrome 1 and Cryptochrome 2

To ensure that *MagR* expression was not masked by the indirect effects of knocking down one *Cry* on the expression of the other, a double knockdown of *Cry1/2* was executed.

**Figure 5:**
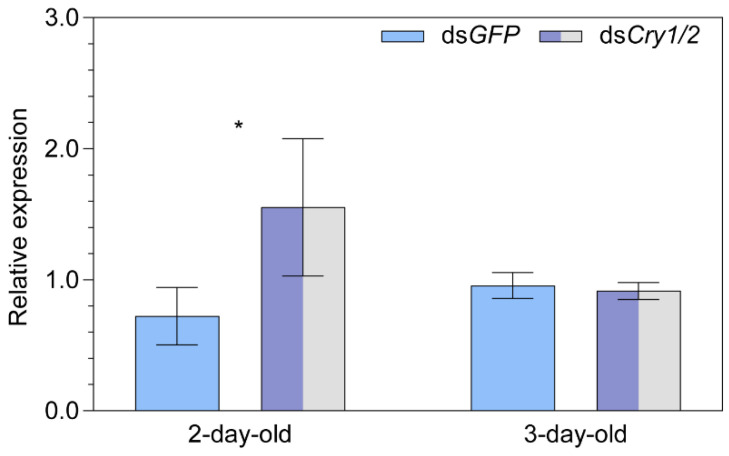
The relative transcript abundance of *Iron-sulfur Cluster Assembly 1* (i.e., *MagR*) in two- and three-day-old female adult *Nilaparvata lugens* after injection of both ds*Cry1* and ds*Cry2*. Statistical significance of differences in *MagR* expression between ds*Cry1/2* treatment and ds*GFP* control groups is tested using one-way ANOVA at *p* < 0.05 (*).

A significant upregulation of *MagR* was found for 2-day-old females (+115.0%, *p* = 0.026) in the ds*Cry1/2* vs. ds*GFP* group, while the difference was negligible in 3-day-old females (Figure 5).

## 3. Discussion

The enigmatic mechanisms underlying magnetoresponse, especially radical-pair-based magnetoreception or magnetosensitivity, have been extensively explored across various taxa and serve as a major area of study in quantum biology [4,6,7,9,11,70]. Recently, MagR (originally named IscA1)/Cry complex magnetosensing [15,16,17] has expanded the canonical Cry-involved radical-pair magnetoreception model, and is supported by increasing evidence [16,25,26,36,39,45,48,49,52]. Using a reverse genetic assay, we show for the first time in vivo transcriptional crosstalk between *Crys* and *MagR* in the migratory brown planthopper, *N. lugens*, genes that may enable its magnetoresponse to both physiological and non-physiological changes in MF intensity. The magnetoreception and orientational abilities of migrating birds are well known, and birds most likely possess both cryptochrome-based and magnetite-based sensing systems [70]. It is intriguing to note that a seemingly simple invertebrate like *N. lugens* studied here has also seemingly developed such a complex magnetoresponse, likely as a result of long-term evolution driven by selection for traits that confer a migratory advantage.

We replicated the findings of our previous study [61], which demonstrated an increase in both *Cry1* and *Cry2* expression in *N. lugens* when exposed to the GMF_45μT_ vs. GMF_50μT_. Consistent with the upregulation of *Cry1* in nymphs under the NZMF vs. GMF_50μT_ [62], the transcript expression of *Cry1* in female adults was also found upregulated in the present study. However, the comparison of *Cry2* magnetoresponse between nymphs in [62] and adults in this study under NZMF vs. GMF_50μT_ suggests a temporal-specific transcriptional magnetoresponse consistent with its multifunctional role as a circadian repressor, exhibiting rhythmic expression [66]. The involvement of *Crys* in the magnetoresponse of *N. lugens*, as implicated here, is consistent with prior transgenic research on the magnetoreception system of *Drosophila* [54].

In contrast to *Crys* expression patterns, the *N. lugens* treatment group exposed to physiological magnetic fields showed a general increase in *MagR* transcript levels, while no such increase was observed in the NZMF vs. GMF_50μT_ group, implying a narrower threshold for magnetoresponsiveness of *MagR*. It is interesting to note that *Cry1* appears to hold greater importance or at least sensitivity as a magnetoreceptor or magnetotransducer, given that significant magnetoresponses were observed in all 1- to 3-day-old adults under physiological changes in magnetic field intensity while changes in the expression of the other two genes examined were less extensive. Notably, all three putative magnetosensing genes exhibited a greater overall relative increase in expression levels in the physiological MF group compared to the non-physiological one, consistent with their potential roles in magnetoreception during normal insect migration.

In vitro studies across taxa have shown that Cry and MagR proteins interact to form a rod-like polymeric complex with an intrinsic magnetic moment [15]. However, limited in vivo information is available regarding their potential interplay. Knockdown of *MagR* in *Drosophila* has been reported to disrupt circadian behavior, indicating its involvement in the circadian pacemaker [34]. Consistent with the findings in *Drosophila*, our study has observed that knocking down *MagR* affects the expression of multifunctional *Cry1*, which not only acts as a putative magnetoreceptor but also plays a role as the photoreceptor responsible for synchronizing the circadian clock [6,19,20,66]. Our single gene knockdown assay did not reveal any transcriptional interactions between *Cry2* and *MagR*, which is consistent with the results of our protein interaction investigations using a yeast two-hybrid assay in *N. lugens* [39]. This finding also supports the suggested Cry/MagR complex magnetoreception model [15], which proposes that the Cry should be photosensitive like Cry1 here.

The interplay between Cry1 and Cry2 has been insufficiently explored to date across taxa. In our study of *N. lugens* here, reciprocal knockdown experiments resulted in significant changes in both *Cry1* and *Cry2* in 2- and 3-day-old adults. This can be attributed to their involvement in the circadian pathway, where Cry1 functions as a blue-light circadian photoreceptor that resets the molecular clock upon light exposure, while Cry2 acts as a transcriptional repressor in the clockwork [71]. Interestingly, while the individual knockdown of *Cry1* or *Cry2* did not affect the transcriptional expression of *MagR*, the double knockdown of both significantly upregulated *MagR* expression in 2-day-old adults, which could be due to a complementary effect between *Cry1* and *Cry2*. Thus, *Cry1* and *Cry2* seemingly act in concert as the repressor of *MagR* expression, although the involvement of unknown gene (s) such as opsins [50,72] cannot be excluded. As 2- and 3-day-old adults correspond to the primary age for long-distance migration takeoff and termination [68], both *Crys* and *MagR* may have potential signaling roles in initiating and terminating migration. However, further investigation is required to elucidate the specific mechanism and functional significance for migration.

**Figure 6:**
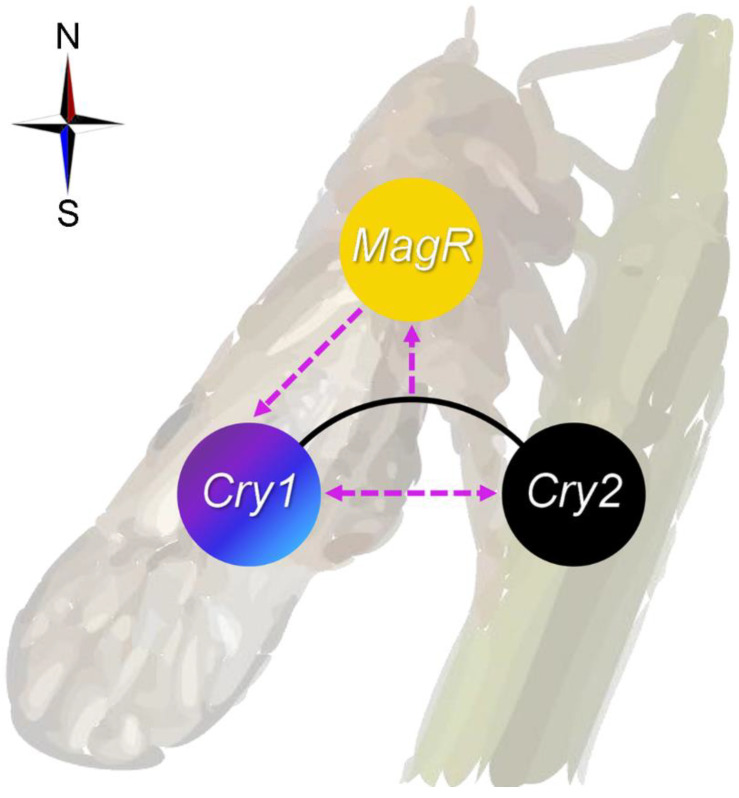
Proposed transcriptional crosstalk between *Cryptochromes* (*Cry1 and Cry2*) and *Iron-sulfur Cluster Assembly 1* (*MagR*) in magnetoresponse of the migratory brown planthopper, *Nilaparvata lugens*. The potential transcriptional interactions, whether activating or inhibiting, are indicated by dashed lines with an arrow. The compass represents that the crosstalk is proposed within the context of magnetoresponse.

Compared to non-migratory insects [7,56,57,73], insect migrants appear to possess a higher degree of magnetosensitivity in response to changes in MFs, which is consistent with their potential utilization of MF information during their long-distance movements [61]. Therefore, they hold great promise for providing unique insights into magnetoresponse mechanisms. A recent study has demonstrated that free FAD itself can mediate a magnetoresponse in vivo, albeit at high levels that are non-physiological, suggesting a possible shift from Cry-centric radical-pair mechanism to a non-Cry-dependent model [7,27]. Considering this, Cry1, Cry2 and MagR of the migratory *N. lugens* may act in concert (as proposed in Figure 6) as the magnetotransducer due to their reported involvement in animal migration or magnetoresponses [6,7,24,25,26,67]. An improved understanding of these interactions may also provide useful in the optimization of bionic magnetosensors based on the MagR/Cry complex [46,47]. Lastly, as a note of caution, given that our work has clearly depicted a transcriptional interplay among three genes involved in magnetoresponse, particularly between *Cry1* and *MagR*, careful thought should be used when conducting functional genetic analyses of magnetoresponse to avoid potential bias resulting from their crosstalk.

## 4. Materials and Methods

### 4.1. Insects

The brown planthopper *N. lugens* were initially gathered from paddy fields located in Nanjing, Jiangsu province of China, in summer. They were kept indoors and fed on Taichung Native 1 rice seedlings, with a 14-h light and 10-h dark cycle, at a temperature of 26 °C and relative humidity between 70% and 80%. All subsequent experiments were conducted under these same environmental conditions, with the exception of the magnetic field variations. Before being assigned to the magnetic field experimental groups, the colony was maintained under the local geomagnetic field conditions (~50 μT).

### 4.2. Exposure of Insects to Magnetic Fields

In this study, we used two three-axis DC-type Helmholtz coil systems with an external diameter of 1200 mm to mimic the local geomagnetic field (50,000 ± 307 nT; i.e., GMF_50μT_) at Nanjing city (32°3′42″ N, 118°46′40″ E) vs. the near-zero magnetic field (NZMF) (519 ± 32 nT) and GMF_50μT_ vs. the GMF intensity of another point on the migration route of *N. lugens*, Zhanjiang city (21°12′29″ N, 110°21′11″ E; mimic intensity: 45,000 ± 233 nT; i.e., GMF_45μT_), at a similar inclination and declination within the effective homogeneous areas of 300 × 300 × 300 mm (<2% heterogeneity), as described before [61,64]. A Faraday cage was placed inside each coil to shield the insects from potential anthropogenic electromagnetic noise. We measured and adjusted the magnetic field parameters daily using a fluxgate magnetometer (Model 191A, HONOR TOP Magnetoelectric Technology Co., Ltd., Qingdao, China) and ensured that both groups were in the same room with uniform environmental factors except for magnetic fields.

During the study, we exposed *N. lugens* raised separately in glass tubes (diameter: height = 3.0 cm: 15 cm) to two distinct treatments: GMF_50μT_ vs. GMF_45μT_ and GMF_50μT_ vs. NZMF. This exposure began from mated F0 females up to unmated 1- to 3-day-old F1 adults, following the protocol outlined in our previous study [74], with the addition of a quick dsRNA injection. After sampling at the same time point under corresponding magnetic conditions, individuals were quickly euthanized in liquid nitrogen for total RNA isolation.

### 4.3. RNA Isolation and cDNA Synthesis

Four biologically independent pools were used, each containing five female adults for each group based on their developmental stage, dsRNA injection, and magnetic field intensity. Total RNA was extracted from these pooled samples using TRIzol^®^ (Invitrogen; Thermo Fisher Scientific, Inc., Waltham, MA, USA). The extracted RNA samples were individually analyzed for quality and quantity using a NanoDrop2000 (Thermo Fisher Scientific, Inc., Waltham, MA, USA). Prior to reverse transcription, the integrity of each total RNA sample was assessed by electrophoresis in 1% agarose gels. Subsequently, cDNA was synthesized from 500 ng of total RNA in a 20 μL reaction, utilizing the PrimeScript RT reagent kit supplemented with a gDNA Eraser (Takara Bio Inc., Dalian, China).

### 4.4. Double-Stranded RNA Preparation

The gene sequences of potential key magnetoreception genes *Cry1*, *Cry2*, and *MagR* in *N. lugens* were obtained from our previous studies [38,66]. To minimize the occurrence of off-target RNA interference (RNAi), the specific regions of the target genes selected for double-stranded RNA (dsRNA) synthesis were carefully verified to ensure that there were no other matches in the transcriptome and genome databases of *N. lugens*. A green fluorescent protein (GFP) gene (GenBank: MF169984.1), which is an exogenous gene to *N. lugens*, was utilized as the control (ds*GFP*) [75]. Their specific primers used to synthesize the dsRNA containing the T7 promoter sequence were designed using Oligo 7 software (Molecular Biology Insights, Inc., Cascade, CO, USA) (Table S1, underlined sequence indicates T7 promoter). The dsRNA was synthesized using the T7 RNAi Transcription Kit (Vazyme Biotech Co., Ltd., Nanjing, China) according to the manufacturer’s instructions. To purify the dsRNAs, 50 μL of 95% ethanol and 2 μL of 3 M sodium acetate (pH 5.2) were added, followed by washing with 70% ethanol. After drying, the dsRNA was resuspended in an appropriate amount of nuclease-free water. The concentration and quality of dsRNAs were determined by NanoDrop 2000 (Thermo, Waltham, MA, USA) and gel electrophoresis analysis (1%), respectively. All dsRNAs were then adjusted to a concentration of 4500 ng/μL and stored at −80 °C for future use.

### 4.5. Microinjection of dsRNA

Fourth-instar *N. lugens* nymphs were subjected to low-temperature anesthesia, and the immobilized nymphs were gently transferred onto an injection plate using a soft brush. Using a Micro4 microinjection apparatus, 40 nL of purified dsRNA with a concentration of 4500 ng/μL was slowly injected into the outer epidermis of the thoracic and hind legs of *N. lugens*. After injection, the nymphs were placed back and maintained separately in glass tubes.

### 4.6. Transcript Expression Analysis

The potential key genes in magnetoreception signaling including *Cryptochrome 1* (*Cry1*), *Cryptochrome 2* (*Cry2*) and *Iron-sulfur Cluster Assembly 1* (i.e., *MagR*) were selected for transcript expression analysis using quantitative real-time polymerase chain reaction (qRT-PCR) assay. The qRT-PCR was conducted on an Applied Biosystems^®^ QuantStudio™ 5 Flex Real-Time PCR System (Thermo Fisher Scientific, Inc., Waltham, MA, USA) using SYBR Premix Ex Taq (Tli RNaseH Plus; Takara Bio Inc., Dalian, China). The reactions were conducted in a final volume of 20 μL (including 2 μL of a 1/20 dilution of the cDNA template and primers in a final concentration of 200 nM) with the following conditions: an initial 30 s step of 95 °C followed by 40 denaturation cycles at 95 °C for 5 s and primer annealing at 60 °C for 34 s. The *EF1-α* and *RPL5* were used as the reference genes [64], and the 2^−∆∆Ct^ method (Ct, cycle threshold) was applied to evaluate the relative expression levels [76]. Four biological replicates were used for statistical comparison between groups.

### 4.7. Statistical Analysis

All data were analyzed using SPSS 26 (IBM Inc., Armonk, NY, USA). The Shapiro–Wilk test was used to test for normality (*p* > 0.05) and Levene’s test for the homogeneity of variances (*p* > 0.05), before an analysis of variance (ANOVA). The one-way ANOVA or a two-tailed nonparametric Mann–Whitney *U* test (if *p* ≤ 0.05 for normality or homogeneity of variances test) was used to test for the effect of magnetic field intensity or gene knockdown on the transcript relative expression levels of *Cry1*, *Cry2* and *MagR* at α = 0.05.

## Data Availability

Not applicable.

## Supplementary Materials

The following supporting information can be downloaded at: https://www.mdpi.com/article/10.3390/ijms241311101/s1.
