# Supplementary material for: Probing Transcriptional Crosstalk between Cryptochromes and Iron-sulfur Cluster Assembly 1 (MagR) in the Magnetoresponse of a Migratory Insect
Source: Int J Mol Sci. 2023 Jul 5;24(13):11101. doi: 10.3390/ijms241311101 (PMC10342043; doi:10.3390/ijms241311101)
Supplement: Supplementary file 1 [file ijms-24-11101-s001.zip › ijms-2454938-supplementary.pdf]

Supplementary information

Supplementary figure

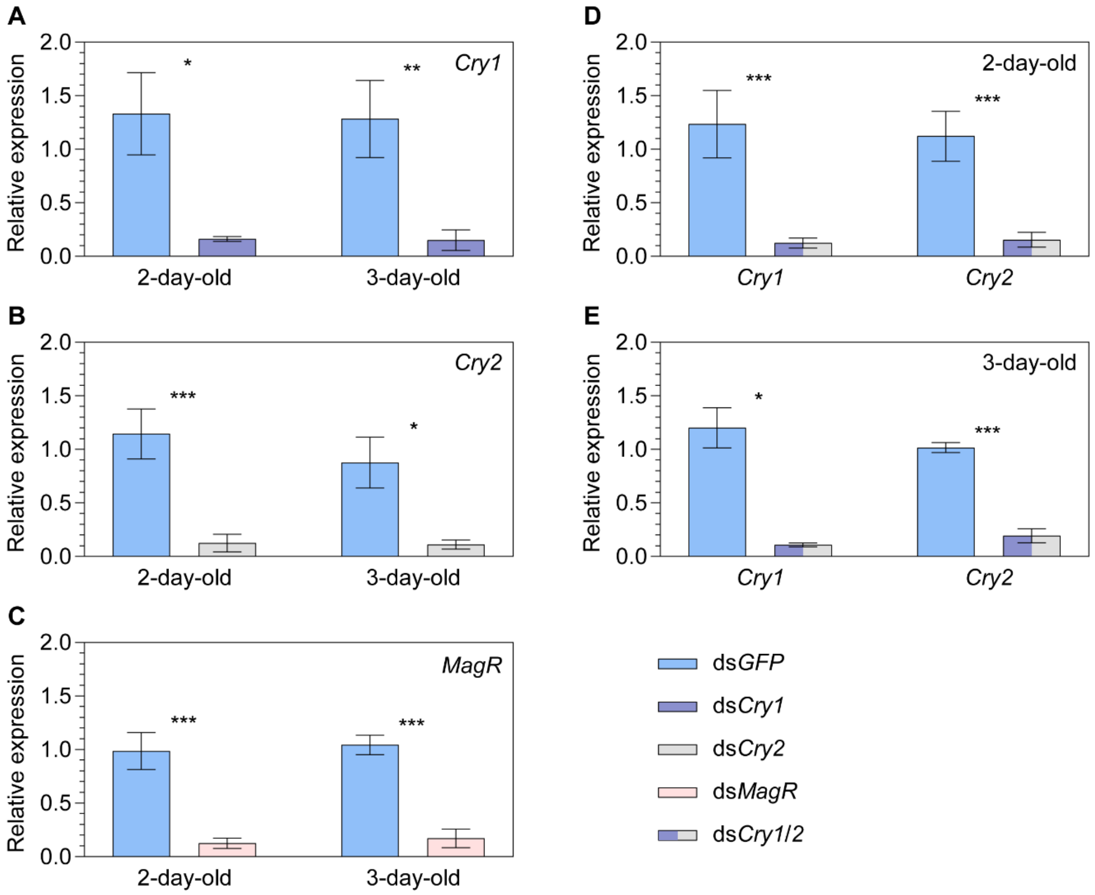

Figure S1. Examination of RNAi efficiency by qRT–PCR. The injection of dsRNA was performed one day before adult eclosion, and the relative transcript abundance of each gene was normalized to the expression level of *NlRPL5* and *NlEF1-α* for two- and three-day-old female adults. Four biologically independent pools, each containing ten adults, were used. The columns represent averages with vertical bars indicating standard errors. Statistical significance of differences in expression between the dsRNA injection of the target gene and *GFP* groups is tested using one-way ANOVA at $P < 0.05$ (*), $P < 0.01$ (**) and $P < 0.001$ (***).

Supplementary table

Table S1. The Primers used in this study.

**Primer name**	**Sequence*** **(5′ to 3′; F, forward; R, reverse)**	**Purpose**
ds*MagR*	F: TAATACGACTCACTATAGGGAGAAAGGAAAGTTTGACGAAG	*Iron-sulfur Cluster Assembly 1*
	R: TAATACGACTCACTATAGGGAGCCCTAAATATTAACATCGT	*(i.e., MagR)* dsRNA synthesis
ds*Cry1*	F: TAATACGACTCACTATAGGGGCCAATCACAACTTCTATCC	*Cryptochrome 1* dsRNA synthesis
	R: TAATACGACTCACTATAGGGACTCCTGGGTACAGTAATATG	
ds*Cry2*	F: TAATACGACTCACTATAGGGCATCCAATGTCAGCATCAAT	*Cryptochrome 2* dsRNA synthesis
	R: TAATACGACTCACTATAGGGCAATAAGCCTTCTGTGTCAA	
ds*GFP*	F: TAATACGACTCACTATAGGGATGGTGAGCAAGGGCGAGGAG	*GFP* dsRNA synthesis
	R: TAATACGACTCACTATAGGGCGGATCTTGAAGTTCACCTTG	
q*MagR*	F: ATCCCAACATAAAAGGTACTTGC	qRT-PCR for *MagR*
	R: ATGGTAGCCCTAAATATTAACATCG	
q*Cry1*	F: CGTAGAGCACAAAGTCGCTTC	qRT-PCR for *Cry1*
	R: CCACATGAATTGGCGTACCTC	
q*Cry2*	F: ACAAACCTGACGTTCGAGGA	qRT-PCR for *Cry2*
	R: TAGAGAGTATGCGACACCCTT	
q*RPL5*	F: GACCAATTATGCCTCAGCCTAC	Reference gene used in qRT-PCR
	R: CAGAGCCTCCACATTGTACTCC	
q*EF1-α*	F: ATCAGCCATTCAACTCACCTCC	Reference gene used in qRT-PCR
	R: AACACGACGATACATGCGATAC	

* Including published primer sequences in our previous work.
